# Spatial patterns of transcriptional activity in the chromosome of *Escherichia coli*

**DOI:** 10.1186/gb-2004-5-11-r86

**Published:** 2004-10-27

**Authors:** Kyeong Soo Jeong, Jaeyong Ahn, Arkady B Khodursky

**Affiliations:** 1 Department of Biochemistry, Molecular Biology and Biophysics, University of Minnesota, St. Paul, MN 55108, USA

## Abstract

**Background:**

Although genes on the chromosome are organized in a fixed order, the spatial correlations in transcription have not been systematically evaluated. We used a combination of genomic and signal processing techniques to investigate the properties of transcription in the genome of *Escherichia coli *K12 as a function of the position of genes on the chromosome.

**Results:**

Spectral analysis of transcriptional series revealed the existence of statistically significant patterns in the spatial series of transcriptional activity. These patterns could be classified into three categories: short-range, of up to 16 kilobases (kb); medium-range, over 100-125 kb; and long-range, over 600-800 kb. We show that the significant similarities in gene activities extend beyond the length of an operon and that local patterns of coexpression are dependent on DNA supercoiling. Unlike short-range patterns, the formation of medium and long-range transcriptional patterns does not strictly depend on the level of DNA supercoiling. The long-range patterns appear to correlate with the patterns of distribution of DNA gyrase on the bacterial chromosome.

**Conclusions:**

Localization of structural components in the transcriptional signal revealed an asymmetry in the distribution of transcriptional patterns along the bacterial chromosome. The demonstration that spatial patterns of transcription could be modulated pharmacologically and genetically, along with the identification of molecular correlates of transcriptional patterns, offer for the first time strong evidence of physiologically determined higher-order organization of transcription in the bacterial chromosome.

## Abstract 2

Analysis of the transcriptional activity in *Escherichia coli* K12 revealed an asymmetry in the distribution of transcriptional patterns along the bacterial chromosome and showed that spatial patterns of transcription could be modulated pharmacologically and genetically.

## Background

Chromosomes have evolved to effectively retrieve and transmit genetic information stored in DNA. Significant progress has been made recently in our understanding of how DNA is packaged into chromosomes, particularly at low compaction levels determined by supercoiling and/or protein-dependent condensation [1]. Available structural information about bacterial chromosomes indicates that the chromosome is supercoiled *in vivo *[2] and organized in topologically constrained domains [3]; diffusion of supercoils over the chromosome is impeded in actively replicating cells [4]; the chromosome is mildly condensed *in vivo *[5,6]; chromosomal loci inside the cell are specifically organized and arrayed in linear order according to the linear genetic map [7,8]; and at least two chromosomal loci are actively moved and positioned inside the cell [7,9].

Although the molecular bases of these structural features are not known, the bacterial chromosome can be viewed as a cellular organelle, whose dynamics may be coupled to the state of the cell. In turn, the state of the cell is reflected in the whole-genome transcriptional activity [10]. Therefore, genome-wide transcription can be used to probe chromosomal organization. Global transcriptional profiles have been successfully used to probe the organization of transcriptional units into operons [11] and regulons [12,13]. However, such analysis is limited by assumptions about the nature of transcriptional units.

Covariation in transcriptional activity along the chromosome determine spatial transcriptional patterns [14,15]. Such co-variation might result from differing DNA accessibility along the chromosome [16,17]. The variation in accessibility, in turn, may be determined by chromosomal structural features. By analogy, chromosomal regions that do not reveal any spatial covariation could represent unstructured portions of the chromosome.

Using signal processing and statistical techniques, we systematically examined transcriptional activity of genes as a function of their position in the bacterial chromosome. Here we report the discovery of stable, short- and long-range patterns in genome-wide transcription in *Escherichia coli *K12. Moreover, we demonstrate that such patterns are affected by genetic and environmental factors, thereby offering the first biologically relevant insights into the nature of the spatial organization of transcription in bacteria.

## Results

### Local structure in the spatial series of transcriptional activity

**Figure 1:**
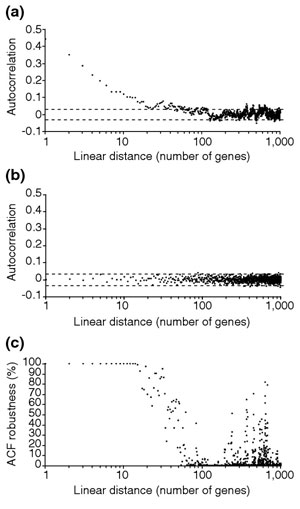
The autocorrelation function (ACF) of the spatial series of transcript abundances. **(a) **ACF of the series recorded in LB medium. **(b) **ACF of the series after scrambling the linear order of genes. The dotted line delimits the 95% confidence band. **(c) **The robustness of autocorrelations in the series recorded in LB as determined by bootstrapping. The robustness is expressed as a percentage of times the autocorrelation at the corresponding lag satisfied the critical value threshold (α = 0.05) following bootstrapping of the observed relative abundances. The sampling was done 1,000 times.

We modeled transcriptional activity of the chromosome as a one-dimensional spatial series of transcript abundances. Transcript abundances were measured in cells grown in batch cultures to OD_600 _= 0.5 in LB or M9 medium supplemented with 0.2% glucose (cultures reached stationary phase at OD_600 _= 3.5 in LB medium and at 2.5 in M9 medium). Samples of total RNA extracted using hot-phenol method [18] were labeled with Cy-fluorophors and hybridized against genomic DNA as a reference. We carried out two types of hybridization: one using genomic DNA from the same cells from which we extracted the RNA and the other using genomic DNA from cultures with arrested initiation of DNA replication that completed ongoing rounds of replication. Two different types of genomic reference produced indistinguishable results in spectral analysis, and for the sake of simplicity we present here the analysis of results obtained in hybridizations against genomic DNA isolated from non-replicating cultures. The mRNA abundances have been recorded for almost every gene in the chromosome by two-color hybridization on whole-genome DNA microarrays. The data are publicly available at the Gene Expression Omnibus [19], accession numbers GSE1730 and GSE1735. To determine the degree of similarity in transcriptional activity of individual genes as a function of their position on the chromosome we calculated the autocorrelation function (ACF) as a function of the distance between genes, where the distance is measured as the number of intervening genes (Figure 1).

Independent of the growth conditions, the ACF could be characterized as a decaying function whose largest statistically significant portion assumes positive values and corresponds to relatively short gene-to-gene distances (fewer than 100 genes; Figure 1a, data not shown). By definition [20], the portion of the ACF that constitutes significant correlations may reflect the existing stable structure in the series. Thus, it is likely that the transcription of any two genes, separated on the chromosome by a distance less than or equal to the length of the structured portion of the series, is similarly affected on average across the entire chromosome. Scrambling the linear order of genes leads to the loss of structure in the series (Figure 1b), indicating that the relative positioning of genes determines the autocorrelation properties.

If the autocorrelation reflects dominant patterns of co-transcription, then by determining the properties of the ACF we should be able to describe these patterns. Any one-dimensional pattern could be characterized by the stability (the distance between two genes at which transcriptional similarity drops to 50% of the average correlation between immediate neighbors) and by the range of stable correlations (maximal length of an average stable co-transcribed chromosomal region). Following the two-parameter exponential fitting of at least eight independently measured ACFs, we determined that the transcriptional correlations decay by half in the rich medium over 7.5 kilobases (kb) (Table 1, 0 minute entry).

The range of local transcriptional patterns was measured as the maximal length of a continuous, significantly correlated region of the chromosome that is not affected by experimental error. We determined that in rich medium up to 16 genes in a row could demonstrate apparently coherent transcriptional activity (Figure 1c). The short-range correlations of the transcriptional series obtained in minimal medium had comparable characteristics (data not shown). While it is expected that genes organized in operons would show significant autocorrelation, the stability as well as the significant range of autocorrelations observed here extend far beyond those expected if gene expression was only coordinated within operons, which have an average size of three genes [21].

### Analysis of long-range correlations

**Figure 2:**
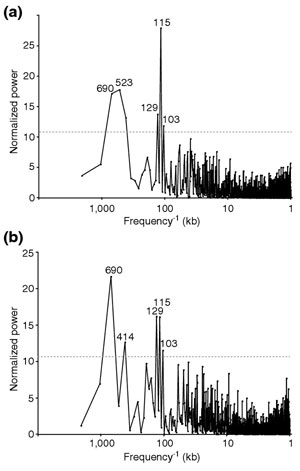
The Lomb periodogram of transcriptional activity. Wild-type cells in **(a) **LB or **(b) **M9 medium. The significant frequencies at the 95% confidence level (dashed line) are indicated by their scales in kilobase-pairs.

In addition to continuous transcriptional correlations over short distances, we also observed individual, statistically significant spikes in correlations of transcriptional activities of genes located about 100 and 700 kilobases (kb) apart. To investigate such long-range transcriptional patterns in more detail we decomposed the original signal of transcript abundances into a series of harmonics. The frequency spectrum of transcript abundances is shown in Figure 2. Four frequencies appear to be significant (at 95% confidence level) in the spatial series obtained from the cultures grown in LB medium: 690^-1 ^kb^-1^; 129^-1 ^kb^-1^, 115^-1 ^kb^-1 ^and 103^-1 ^kb^-1^. The sequential signal recorded in the mid-exponential cells grown in M9 salts supplemented with glucose contained similar significant frequencies (Figure 2b): 690^-1 ^kb^-1^, a clump of frequencies around 115^-1 ^kb^-1 ^and a free-standing frequency of 414^-1 ^kb^-1^.

**Figure 3:**
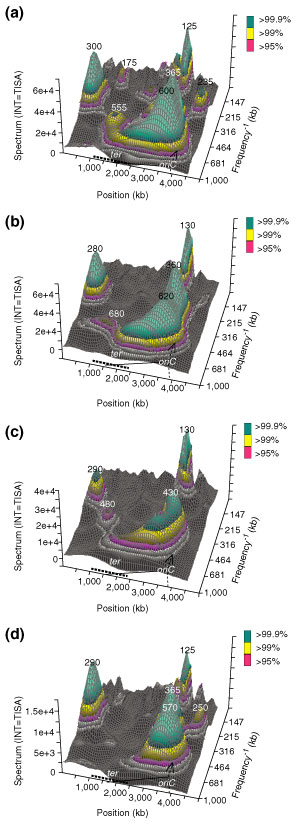
Three-dimensional (3D) wavelet power spectrum of the transcriptional signal. The wavelet power spectrum from 100 to 1,000 kb in wild-type *E. coli *cells grown in LB and treated with norfloxacin at 10 μg/ml for **(a) **0 min, **(b) **10 min, **(c) **30 min and **(d) **in the *gyrA *(D82G) mutant grown in LB without a drug. The analysis was performed in AutoSignal 1.6 using the Morlet wavelet as the basis with the randomized set showing no significant pattern at 95% (magenta) confidence level. The significant wavelet components are represented as parts of a power spectral density (PSD) plot, where power is calculated as the space (time in standard notations)-integral squared amplitude (TISA). Our model assumes white background noise.

The Fourier transform provides signal average characteristics and does not determine the frequencies at a particular spatial locality. To localize significant frequencies determined by Fourier transform and to find potentially significant local spikes of transcriptional activity, we subjected the spatial series of transcript abundances to a wavelet transform. The wavelet analysis revealed significant spectral components at the scales very similar to significant periods in the Fourier spectrum: approximately 125 kb and 600-700 kb (Figure 3a). In addition, in the range of scales from 100 to 1,000 kb, the wavelet transform identified pronounced local patterns at frequencies corresponding to 235^-1 ^kb^-1^, 300^-1 ^kb^-1^, 365^-1 ^kb^-1 ^and 555^-1 ^kb^-1^. The wavelet spectrum also shows that patterns of transcriptional activity are not symmetrical with respect to the chromosome. The dominant patterns are localized largely in the left replichore (the half chromosome divided by the replication axis counter-clockwise from the *oriC*) and appear to be bounded by the origin of replication. The most dominant pattern wave in transcriptional series, represented by a period of about 600 kb, spreads for 2.3 megabases (Mb), from the origin of replication to the *terC *site. The second most pronounced pattern (around 125 kb) consistent with the Fourier spectrum results from the transcriptional activity between the origin of replication and *terG*, about 1.3 Mb away. While significant components of the wavelet spectrum consistent with the Fourier spectrum were largely distributed on the left replichore, the scales unique to the wavelet spectrum were narrowly distributed along the scattered parts of the right replichore.

### Modulation of transcriptional spectra

**Figure 4:**
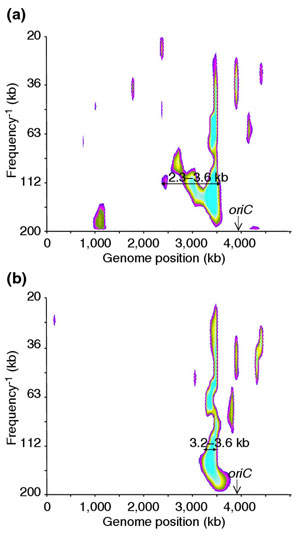
Two-dimensional (2D) wavelet power spectrum of transcriptional activity in the vicinity of the origin of replication. The wavelet power spectrum from 20 to 200 kb of transcriptional activity of wild-type *E. coli ***(a) **untreated and **(b) **treated with norfloxacin for 30 min. The 2D wavelet spectrum was calculated as in Figure 3.

**Figure 5:**
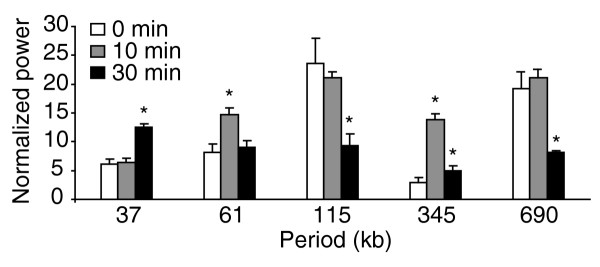
Comparison of significant frequencies from the Fourier spectra in the cultures treated with norfloxacin. The amplitudes of the most significant frequencies have been compared using the two-tailed Student's *t*-test; the asterisks mark the frequencies whose power was significantly different (*p *< 0.05) from the power of the corresponding frequencies in the spectrum from the untreated cells (0 min).

Inhibition of transcription initiation by rifampicin completely eliminates significant frequencies from the spectra after 30 min of treatment (data not shown). We rationalized that if transcription is not only inhibited, but modulated, globally, we might be able to track changes in the transcriptional spectra. We used a topoisomerase inhibitor, norfloxacin, to try to modulate transcription in the cell by inhibiting topoisomerase activities. As supercoiling and transcription in the cell are expected to be tightly coupled [22], we anticipated that inhibition of DNA topoisomerization would affect spatial transcriptional patterns. Norfloxacin was used at a concentration that ensured 50% and 90% killing of a bacterial population after 10 minutes and 30 minutes treatment, respectively. We examined the local correlations in transcription following norfloxacin treatment and found that by 30 minutes the range of significant autocorrelations has been reduced by about 50% from 16.5 to 7.7 kb and the stability of local patterns was reduced from 7.5 to 4.3 kb (Table 1). We have observed changes in the amplitude (the magnitude of squared amplitudes integrated over space - the power - is plotted along the vertical axis in Figure 3) as well as in spatial ranges of significant wavelet components (Figure 3b,c). The most obvious changes in the wavelet spectrum can be described as a recession of the largest-scale wavelets from the terminus of replication in the left replichore. At a higher resolution, as seen in the local wavelet power spectrum in Figure 4, the spatial range of the characteristic 100-125 kb wavelet narrowed by 25 to 90%. Similarly, the Fourier analysis revealed that the main periods in untreated cells, including 115 kb and 690 kb, were significantly (*p *< 0.05) diminished by (in some case) 30 minutes with the drug (Figure 5).

### Global modulation of transcription by a single point mutation in DNA gyrase

In addition to modulation of the transcriptional spectra by non-equilibrium perturbations, we were interested in steady-state alterations in the process of transcription that would not be associated with irreversible changes in bacterial physiology. Such alterations may result from a compensation to a partial reduction in gyrase activity. A transient increase in transcription of the gyrase genes has been observed following inhibition of gyrase activity [23]. We rationalized that a partial loss of function of the gyrase enzyme would be accompanied by a compensatory steady-state increase in transcription of the gyrase genes. We predicted these compensatory changes in transcription would be genome-wide rather than confined to the gyrase genes.

**Figure 6:**
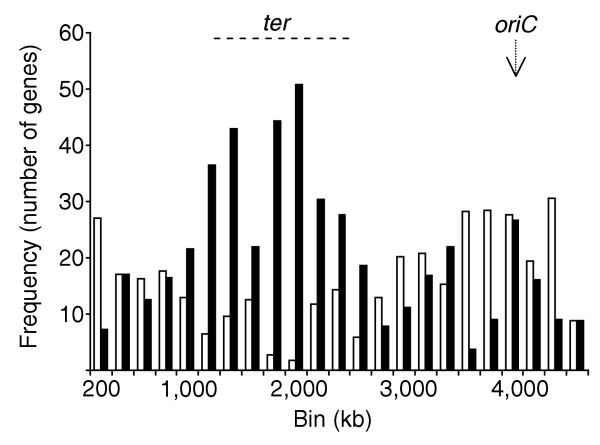
A positional histogram of genes differentially affected in the *gyrA *D82G mutant relative to the wild-type strain. Positional bins of the 487 relatively downregulated genes are shown as filled bars and of the 360 upregulated genes as open bars. The number of genes in each bin are normalized to the bin's size. The sizes of the bins have been determined from the distribution of annotated open reading frames (ORFs) in the *E. coli *genome.

Using resistance to low concentrations of norfloxacin as a screening method, we selected mutants with increased levels of *gyrA *and/or *gyrB *transcripts. We characterized one spontaneous mutant of gyrase, resistant to 0.8 μg/ml of norfloxacin, which carries the D82G mutation in the *gyrA *allele and causes elevated levels of *gyrA *and *gyrB *mRNA *in vivo *and lowers supercoiling activity of DNA gyrase *in vitro *(K.S.J, Hiroshi Hiasa and A.B.K, unpublished work). This mutant had a normal rate of growth and cell density in stationary phase (less than 5% different from the isogenic wild-type strain in LB). As predicted, the compensatory transcriptional mechanism was not limited to transcriptional regulation of gyrase expression but had a global effect. Using microarrays we estimated that steady-state transcriptional activity of as many as 847 genes had changed in the mutant relative to the isogenic wild-type strain. The microarray results were in part confirmed by reverse transcription-PCR (RT-PCR) for 10 out of 10 randomly chosen differentially expressed genes with an observed change in transcript abundance of 50% or greater. The distribution of differentially expressed genes along the chromosome is shown in a histogram in Figure 6. Interestingly, transcription in the area of the chromosome spanning about 1.5 Mb from the vicinity of the *terE *site through the *terG *site appeared to be most affected in the mutant.

If transcriptional activity of the chromosome is differentially perturbed, that is, some regions are being more affected than others, then patterns that existed in unperturbed chromosome may no longer spread across regions with such an 'out of sync' activity, resulting in a partial spatial confinement of originally wider patterns. Moreover, if transcriptional activity is inhibited in a spatial locality, it may cause reduction, or even complete elimination, of local patterns. The wavelet power spectrum in Figure 3d unambiguously confirmed such truncation of wider patterns and the disappearance of significant local patterns in the area of the chromosome where transcription was spatially differentially affected. No changes in transcription have been observed in the gyrase mutant carrying an S83L mutation, a naturally occurring mutation that is not accompanied by a compensatory increase in gyrase transcription (data not shown).

### Spatial patterns of DNA gyrase distribution on the chromosome

**Figure 7:**
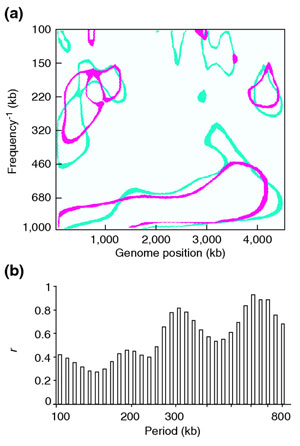
Comparison of the chromosomal spatial patterns of transcriptional activity and of gyrase binding. **(a) **The overlay of the contours of significant components of 2D wavelet power spectra (cyan, transcription activity; magenta, gyrase binding). **(b) ** The dot products of power from the transcriptional and binding spectra at corresponding frequencies.

While global and local patterns of transcription are likely to be due to multiple causes, our data suggest DNA gyrase as an important factor in pattern formation. It has been suggested that DNA gyrase is not randomly distributed along the chromosome of *E. coli *[24]. We examined the distribution of DNA gyrase along the chromosome in a chromatin immunoprecipitation (ChIP) chip assay with GyrA-specific antibodies. The averaged and de-noised spatial signal recorded in eight ChIP-chip microarray experiments were analyzed through a wavelet transform. The contours of significant scales calculated from the gyrase-binding signal and the transcriptional signal obtained under identical growth condition are overlaid in Figure 7a. Similarities between the two spectra were quantified as the dot product of power vectors at corresponding scales (Figure 7b). We observed high positive correlation between the wavelet power spectra of gyrase distribution and transcriptional signal across multiple scales. We found no correlations between wavelet spectra of transcriptional signal and chromosomal distributions of several sequence-specific or nonspecific proteins, including Lrp, Topo IV, FtsK and LexA (data not shown; the results of these ChIP-chip experiments will be summarized in a separate paper).

## Discussion

**Figure 8:**
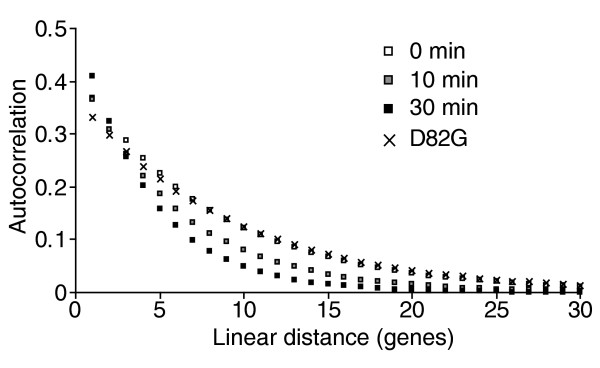
The exponential decay functions fitted by the least-square method into the corresponding autocorrelation functions. The graphs of fitted functions are shown. The goodness of fit was determined by the chi-squared method. Prob_*f*_(χ^2 ^≥ χ^2^_0_) for the ACF functions corresponding to transcriptional series obtained for 0, 10 and 30 min treatment with drug, and for a gyrase mutant were all above 95%.

The development of new high-throughput technologies for parallel analysis of gene expression [25] and the completion of the full *E. coli *genome sequence [26] have enabled us to study the activity of the entire bacterial chromosome simultaneously. Owing to the physical limitations of some techniques and the invasiveness of others, the study of a system is limited to the analysis of signals coming from the system. The bacterial chromosome is a perfect example of a system that cannot be studied directly without interfering with its properties. Therefore, in order to obtain insights into the macroscopic properties of the bacterial chromosome, we chose to study the transcriptional signal that is generated by the chromosome and can be recorded on DNA microarrays. Transcript abundance along the chromosome can be represented as a one-dimensional signal in a spatial domain. The most pronounced feature of this transcriptional signal is a high degree of correlation between genes close together on the chromosome. While such a correlation is expected from genes organized in operons, the observed stability and range of correlations extends far beyond the expected size of the average operon. The stability of the short-range correlations can be significantly reduced by norfloxacin (Table 1, Figure 8) suggesting that short-range correlations depend on negative supercoiling. Such dependence offers an intriguing hypothesis about the physical basis of the short-range transcriptional correlations: the transcription of the genes within a confined supercoiled domain is more similar to each other than to genes in other such domains, with the size of a domain being approximated by the linear stability of the short-range correlations. While this paper was in preparation, Postow *et al*. have shown that the size of a supercoiled domain in the *E. coli *chromosome is of the order of 10 kb [27]. The similarity between the dimensions of supercoiled domains and short-range transcriptional patterns makes our hypothesis even more plausible. Also consistent with our hypothesis is the observation that in a gyrase mutant with a transcriptionally compensated supercoiling function (plasmid DNA supercoiling is normal in the mutant, data not shown) the autocorrelation stability is statistically identical to that in the wild-type cells. By the same argument, however, other characteristics of transcriptional patterns do not appear to be strictly supercoiling-dependent.

The observed changes in the long-range correlations could not be explained by changes in supercoiling because they are observed in the mutant as well as after drug treatment. It is more likely that changes in the medium- and long-range transcriptional patterns are associated with a change in the distribution of gyrase binding to the chromosome. The similarity between patterns of gyrase binding and transcription provides the basis for such a conjecture. It also seems plausible that coherent transcription, or changes in transcription, among clusters of functionally related genes [28] could be associated, in part, with their apparent regular spacing. Co-regulation of such clusters could contribute to the formation of observed local and global transcriptional patterns. We also note that transcription within operons is not sufficient to account for observed short- and long-range patterns. Randomization of the order of operons, as well as of individual genes, completely abrogates both types of correlations. It remains to be seen whether short-range patterns or nonrandom distribution of any sequence features, including extreme secondary structures [29], can contribute to the modulation of long-range correlations.

Analysis of the structure in any signal can be complicated by instrument biases. Such biases in microarray measurements were originally pointed out by Speed and co-workers [30]. Although it has been argued that such systematic effects may preclude adequate spatial-temporal analysis of microarray data [31,32], we offer several reasons why our results are not a microarray artifact. First, the documented patterns could only be observed following subtraction of intensities in the reference DNA channel from the abundance transcript channel and not in the reference channel alone, suggesting that the patterns are not a property of hybridization efficiency. Second, the patterns could be significantly changed. Third, the patterns become more pronounced following the removal of the systematic biases and do not depend on the array design-specific periodicities whose removal by low-pass filtering has no effect on transcriptional patterns; and fourth, modeling of promoter activities carried out by Allen *et al*. [33] revealed the existence of at least one frequency component in the corresponding spatial series that was identical to the lowest-frequency component identified in this study through a direct modeling of transcript abundances.

## Conclusions

This study demonstrates the existence of spatial patterns of transcription in the *E. coli *chromosome. These patterns can be classified on the basis of overall similarity in transcriptional activity of individual genes as well as on the basis of regional similarities. Three major spatial patterns have been identified: short-range correlations that are stable, on average, over 7 kb and could extend up to 15-16 kb; medium-range correlations over 100-125 kb; and long-range correlations over 600-800 kb. These patterns are experimentally stable and can be reproducibly detected in mid-exponential cells grown in batch culture. The growth rate and medium composition appear to have very minimal effects on pattern formation. However, these patterns could be modulated by perturbing DNA gyrase. The significant patterns of gyrase distribution on the chromosome match those of transcriptional activity. Among several proteins (see Results) whose distribution we mapped on the chromosome in mid-exponential culture, the pattern of gyrase binding was the only one coinciding with the patterns of transcription. Although it remains to be seen whether the observed patterns resulted from coherent transcription of functionally related genes regularly distributed along the chromosome and/or through chromosomal organization, the findings presented here are the first evidence of physiologically determined higher-order organization of transcription in any chromosome studied to date.

## Materials and methods

### Strains and culture conditions

All experiments in this study were carried out in *E. coli *K12 strain MG1655 obtained from the ATCC. Mutant *gyrA*^*R *^alleles used in this study carried D82G (this study) and S83L [34] mutations. Wild-type and mutant *gyrA*^*R *^alleles were linked to a Tn*10 *marker and P1 transduced into *E. coli *K12 MG1655 as described previously [35]. MG1655 *dnaC2 *[36] was used to obtain the DNA reference sample for transcript abundance measurements. Bacterial cultures were grown at 37°C in LB or M9 supplemented with 0.2% glucose.

### Microarray analysis

Whole-genome DNA microarrays of *E. coli *were designed, printed and probed as described previously [13,18]. To ensure the success of PCR amplification and to minimize cross-hybridization we redesigned more than 700 primer pairs from the original set of primers supplied by Sigma-Genosys [37]. The relative transcript abundances were determined as described [38]. The RNA samples were extracted from the cultures grown to an OD_600 _of 0.5-0.6 using the hot-phenol method [18]. The experimental error of the measurements of RNA abundances was assessed from at least three independent replicates, where one replicate corresponds to the RNA sample from a bacterial culture grown from a separate colony. Differentially expressed genes were identified using two-class comparisons of the adjusted relative expression values by SAM [39] at 1% false-discovery rate at the 90th percentile. RT-PCR was carried out on ABI Prism 7900 according to the Applied Biosystems protocol with SYBR Green dye as a fluorescent probe.

### Spectral analysis of spatial series

Following the removal of the array-, pin- and dye-specific effects [40,41], the estimated relative abundance values were ordered according to the position of a corresponding gene on the chromosome and subjected to spectral analysis. The spatial domain is defined as a function of the position of the center of mass of the open reading frame (ORF) or operon. In the search for significant frequencies, 2,071 positive frequencies were examined in a signal consisting of 4,143 samples corresponding to individual genes. The autocorrelation function of transcriptional spatial series was calculated as in [20]:



with *j *= 0,1,2,...,*J*, where *y*_*x *_is the series value at the index corresponding to a given ORF location,  is the mean over all *N *observations and *J *is the number of genes in the genome minus 1. The standard error of autocorrelation estimates is determined as (*N*)^-1/2^, where *N *is the number of samples in the series.

Information about the process of transcription could be extracted by identifying a pattern in the observed variations in gene activities followed by a search for the cause or explanation of the pattern. For instance, the pattern may consist of a defined dependence of transcriptional activity as a function of a chromosomal position, or it may consist of the dependence of transcriptional activity as a function of time following a treatment or during the cell cycle. This functional dependence may express itself as a linear variation or harmonic oscillation partially hidden behind the noise. We considered a physical variable *Y *that corresponds to the relative abundance of mRNA. This variable could be measured as a function of the position, *x*, on the *E. coli *chromosome. Values of *Y *are discretely recorded following two-color hybridization on the whole-genome DNA microarrays. Thus there is a finite series of values of *x*, {*x_i_*}, *i *= 1, 2, 3,...., *N *(*N *corresponds to the total number of genes) and corresponding values of *Y*, {*Y_i_*}, *i *= 1, 2, 3,...., *N*. We call {*Y_i_*} a spatial series or sequential series. For the sake of convenience we approximated positions of the centers of mass of individual genes, {*x_i_*}, as evenly spaced. A process is a rule or procedure that generates a sequential series, that is, a prescription for determining the values *Y *for a given set of values of *x*. We defined a spatial series recorded in one whole-genome hybridization as one realization of the process.

The nature of the data that we register using microarrays as well as the nature of the process itself is likely to be such that the rule(s) generating a sequential series specify the probability distribution of {*Y*_*i*_} and not specific values that are the same at every realization. However, defining such a distribution did not seem feasible. Instead, we determined significant autocorrelation components in the spatial series by assessing the effect of experimental error on the significance of correlations. Following the recording of spatial series in at least three independent biological replicates, we simulated realizations of the process of transcription by resampling relative abundances in a gene-specific manner (see [42] for details of the bootstrap). For each of the realizations we calculated the ACF at all acceptable lags. We counted the number of times the ACF value appeared to be significant (α = 0.05) across all simulated realizations at each of the lags.

The Fourier spectrum was determined using the Lomb algorithm [43]. To ensure comparability between the wavelet scale and the Fourier period we used the Morlet wavelet as a basis [44] in Matlab 6.5.1 [45] or AutoSignal 1.6 [46]. The significance of the Fourier and wavelet peaks was estimated from peak-type critical limits. The critical limits were generated from Monte Carlo trials with uniformly spaced spatial domain coordinates. The null hypothesis was simulated using white noise as a background distribution.

### Analysis of gyrase binding on the chromosome

Chromatin immunoprecipitation of DNA sequences bound by DNA gyrase and their detection using whole-genome DNA microarrays were adapted from [47]. Briefly, cells were grown to an OD_600 _of 0.5-0.6 in LB or M9 medium and DNA was cross-linked to proteins with formaldehyde at 1% (v/v) final concentration. Following incubation with monoclonal antibodies against the GyrA subunit of DNA gyrase (TopoGEN), the protein-DNA complexes were precipitated using Protein A-agarose beads (Sigma). Ligation-mediated PCR (LM-PCR; adaptor sequences: 5'-GCGGTGACCCGGGAGATCTGAATTC-3' and 5'-GAATTCAGATC-3') was used to amplify DNA following the reversal of cross-links. Sonicated genomic DNA served as a reference in two-color hybridizations, in which both the sample and the reference were labeled in the Klenow reaction following random amplification by LM-PCR.

## Additional data files

Additional data file 1, available with the online version of this article, consists of a table of the intensities used in the analysis of spatial patterns. The file contains fluorescent intensities recorded in individual channels in two-color microarray hybridizations. The data from replicate arrays are included in the same worksheet, which also contains a brief description of an experiment. The data are also available at [19]; series accession numbers are GSE1730 and GSE1735.

## Supplementary Material

Additional data file 1 A table of the intensities used in the analysis of spatial patterns Click here for additional data file

## Figures and Tables

**Table 1:** Characteristic parameters of the short-range transcriptional patterns

Strain and condition	Stability (kb)	Range (kb)
Wild type, norfloxacin 0 min	7.5 ± 0.9	16.5
Wild type, norfloxacin 10 min	5.6 ± 1.3*	11.0
Wild type, norfloxacin 30 min	4.3 ± 0.9^†^	7.7
*gyrA *(D82G)	8.1 ± 1.1	16.5

*Significantly and ^†^highly significantly different (*p *< 0.05 and *p *< 0.01, respectively) from the stability of the ACF in the untreated sample (0 min), based on the two-tailed *t*-test comparison of the mean fitted parameters for eight independent replicates.
